# Precursor RNA processing 3 is required for male fertility, and germline stem cell self-renewal and differentiation via regulating spliceosome function in *Drosophila* testes

**DOI:** 10.1038/s41598-019-46419-x

**Published:** 2019-07-10

**Authors:** Xia Chen, Xiaojin Luan, Qianwen Zheng, Chen Qiao, Wanyin Chen, Min Wang, Yidan Yan, Bing Xie, Cong Shen, Zeyu He, Jun Zhang, Mingxi Liu, Xing Hu, Hong Li, Bo Zheng, Jie Fang, Jun Yu

**Affiliations:** 1 Department of Gynecology, the Affiliated Hospital of Jiangsu University, Jiangsu University, Zhenjiang Jiangsu, 212001 China; 2 Reproductive Sciences Institute of Jiangsu University, Jiangsu University, Zhenjiang Jiangsu, 212001 China; 3 Department of Clinical Pharmacy, the Affiliated Hospital of Jiangsu University, Jiangsu University, Zhenjiang Jiangsu, 212001 China; 4 Center for Reproduction and Genetics, Suzhou Municipal Hospital, the Affiliated Suzhou Hospital of Nanjing Medical University, Suzhou Jiangsu, 215002 China; 5 Department of Clinical Medicine, China Medical University, Shenyang Liaoning, 110001 China; 6 State Key Laboratory of Reproductive Medicine, Department of Histology and Embryology, Nanjing Medical University, Nanjing, 211166 China

**Keywords:** Differentiation, Spermatogenesis, Self-renewal, Stem-cell niche

## Abstract

The nuclear pre-mRNA spliceosome is a large complex containing five small nuclear ribonucleoprotein particles (snRNPs) and many splicing factors. Messenger RNAs (mRNAs) are generated from pre-mRNAs by the process of RNA splicing, which is conserved in eukaryotes. Precursor RNA processing 3 (Prp3) is a U4/U6-associated snRNP whose function remains largely unknown. In the present study, using genetic manipulation of a *Drosophila melanogaster* testis model, we demonstrated that *Prp3* is essential for male fertility in *Drosophila*. *Prp3* deficiency in germline stem cells (GSCs) and early cyst cells resulted in abnormal structure of testes and maintenance defects of GSCs and cyst stem cells. Knockdown of *Prp3* in spermatogonia and early cyst cells mediated tumor formation caused by differentiation defects. Using an *in vitro* assay, knockdown of *Prp3* decreased proliferation and increased cell death, and controlled the spliceosome function via regulating spliceosome subunits expression in *Drosophila* S2 cells. We also identified two other splicing factors in the Prp complex (*Prp19* and *Prp8*), which mimicked the phenotype of *Prp3* in the *Drosophila* stem cell niche. Our results revealed a significant role of precursor RNA processing factors in male testes, indicating that Prp3, a key spliceosome component in the Prp complex, is essential for male fertility, and germline stem cell self-renewal and differentiation, via regulating the spliceosome function in *Drosophila* testes.

## Introduction

Spermatogenesis is highly conserved and widespread in eukaryotes, from *Drosophila* to humans^1–3^. Mutants of *boule* and many other genes exhibit similar testicular phenotypes in humans and *Drosophila*^4,5^. *Drosophila* works as an excellent animal model to study male fertility^6–8^. Adult *Drosophila* testes contain different stages of germ cells from spermatogonia to mature sperm^8,9^. At the head area of the testis, germline stem cells (GSCs) divide to a new stem cell and a gonialblast, which could proliferate and differentiate into spermatocytes^10^. The gonialblast goes through mitosis process to form a 16-cell spermatogonia cluster, connecting by ring canals and a branched fusome^11,12^. Cyst stem cells (CySCs) can also differentiate to mature cyst cells, which help to maintain the growth of germ cells^3^.

Early germ cells in fly testes are tightly controlled by niche signals^1^. Hub cells secrete the unpaired (Upd) protein, which activates the Janus kinase signal transducer and activator transcription (JAK-STAT) pathway in both GSCs and CySCs^13,14^. Hedgehog (Hh), also secreted in hub cells, is required for CySCs to maintain their pluripotency by Hh signaling pathway^15,16^. Two somatic expressed bone morphogenetic (BMP)-like molecules, Decapentaplegic (Dpp) and Glass bottom boat (Gbb), are essential for the GSC maintenance^17–19^. Bag-of-marbles (Bam) is a significant differentiation factor, which could be repressed by BMP signaling^18,19^. Benign gonial cell neoplasm (Bgcn) is identified as the regulatory factor of Bam, and controls the spermatogonia transition from self-renewal to differentiation^18–20^. Loss of function of *bam* or *bgcn* gene lead to differentiation defects with extensive accumulation of undifferentiated germ cells^20,21^.

Most genes in eukaryotic genomes require the spliceosome to remove the introns from nuclear pre-mRNAs. The major spliceosome (U2 type) and the minor spliceosome (U12 type) are two main types of spliceosomes^22,23^. RNA splicing is conserved and is catalyzed by the spliceosome, containing small nuclear ribonucleoprotein particles (snRNPs) and many non-snRNP protein factors^24^. Comparison of the spliceosome protein composition by mass spectrometry (MS) identified more than 120 proteins in humans and *Drosophila*, indicating the evolutionarily conserved composition of the mRNA spliceosome^25^. Recently evidence demonstrated that U2A, a key component of the spliceosome, is required for male fertility and regulates the transition of germ cells from proliferation to differentiation. A point mutant of human *SNRPA1* in flies also led spermatogonial differentiation defects^26^.

In yeast, precursor RNA processing 3 (Prp3) and several other U4/U6 snRNP-associated splicing genes have been identified by genetic screening for RNA synthesis^27,28^. However, few reports indicate the biological function of Prp3 in animal models. In our previous screen, Prp3 was identified as a male GSC regulatory factor that played roles in *Drosophila* testes^29^. Here, we further investigated roles of Prp3 in male fertility, and the self-renewal and differentiation of germline stem cells in *Drosophila*.

## Results

### Prp3 is crucial for male fertility

**Figure 1:**
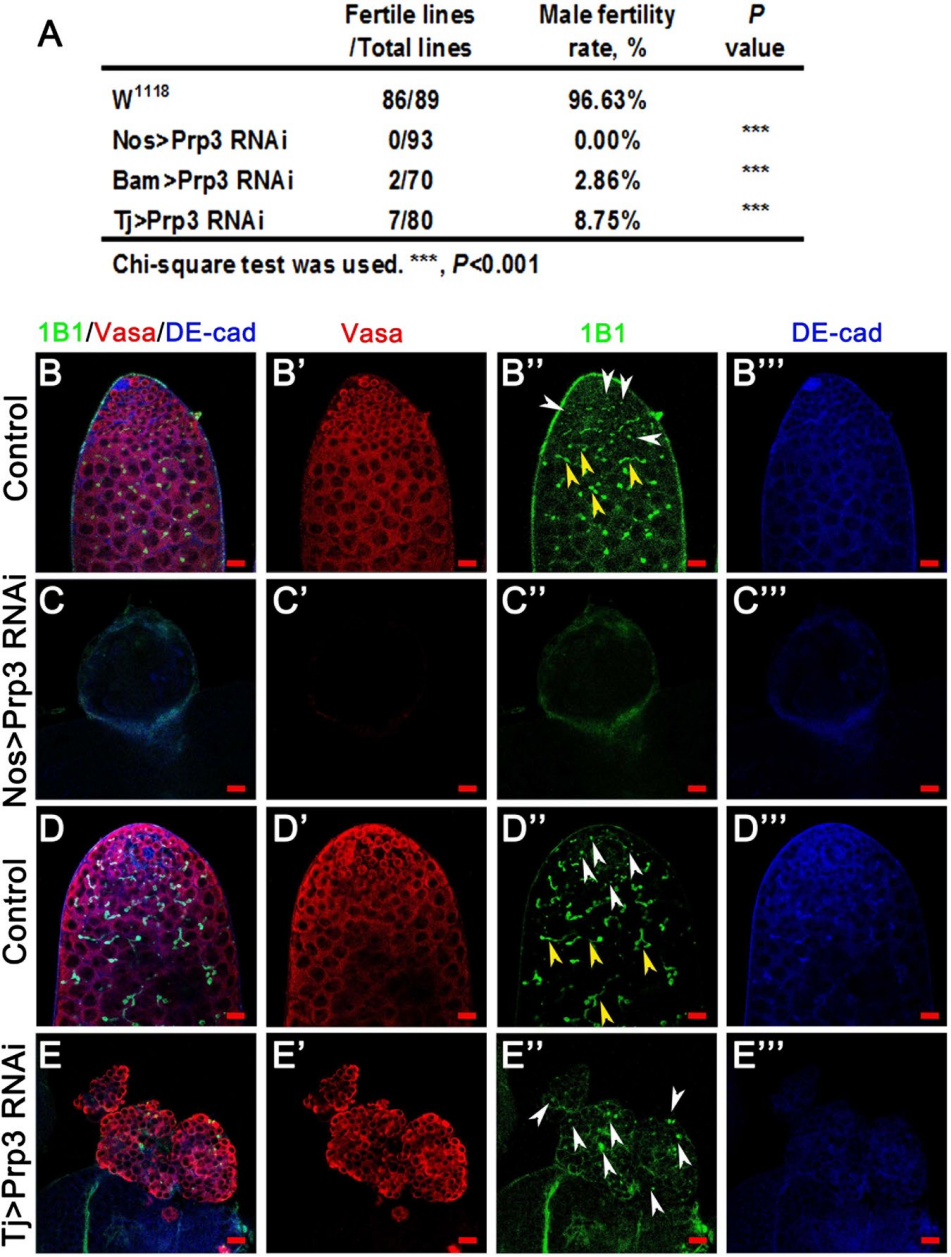
The function of Prp3 in male fertility and the germline stem cell niche. (**A**) The fertility rate of W^1118^ (wild-type), Nos > *Prp3* RNAi, Bam > *Prp3* RNAi, and Tj > *Prp3* RNAi male flies. Chi-square test was used to analyze the results. ***P < 0.001. (**B**–**E**) Immunostaining of control and *Prp3* RNAi testes. Representative point fusomes are indicated with white arrowheads and branch fusomes are indicated with yellow arrowheads. DNA was stained with Hoechst33342. Scale bars: 20 µM.

To explore the role of Prp3 in *Drosophila*, we evaluated the male fertility rate in control and *Prp3* RNAi males. We used three Gal4s to knock down *Prp3* gene: Nos-Gal4 mainly works in early germ cells especially in GSCs, Bam-Gal4 is a spermatogonia driver, and Tj-Gal4 is considered as a cyst cell driver in *Drosophila* testes^29^. When we knocked down *Prp3* driven by Nos-Gal4, males were totally sterile (n = 93) compared with the wild-type control (96.63% fertile, n = 89) (Fig. 1A). We further analyzed the function of the *Prp3* gene by using Bam-Gal4 and Tj-Gal4. Interestingly, males with Bam > *Prp3* RNAi (2.86% fertile, n = 70) and Tj > *Prp3* RNAi (8.75% fertile, n = 80) both lost their fertility ability (Fig. 1A). Taken together, our data suggested that *Prp3* is required for male fertility in *Drosophila*.

### Prp3 is required for GSC maintenance

We next dissected control and Nos > *Prp3* RNAi testes, and stained them with several markers to label the different kind of cells in testes. Somatic cells, including hub cells and cyst cells, can be tagged by DE-cadherin (DE-cad). Fasciclin III (Fas III) labels hub cells, 1B1 labels fusomes in the germ cell cyst and Vasa labels germ cells in the testis. Vasa-positive germ cells closely connected to the hub cells are GSCs and Vasa-negative cells linked together with the hub cells are CySCs^3,30^. In the control testes, point fusomes existed in the early germ cells at the tip of testes and developed into branch fusomes in the differentiated germ cells (Fig. 1B). However, 1B1-positive fusomes and Vasa-labeled germ cells were totally absent in the Nos > *Prp3* RNAi testis (Fig. 1C), which meant that GSCs and differentiated germ cells were not maintained. Our results indicated that Prp3 is required for GSC survival.

### Prp3 is required for CySC maintenance and regulates GSC differentiation

Next, we wondered whether Prp3 plays similar role in CySCs and their differentiated cyst cells. In Tj > *Prp3* RNAi testes, Zn finger homeodomain 1 (Zfh-1)-positive CySCs and eyes absent (Eya)-positive cyst cells were absent when comparing with control testes (Supplementary Fig. 1A,B). Thus, Prp3 is also essential for cell survival of CySCs and mature cyst cells.

Surprisingly, undifferentiated cells accumulated and tumors formed in Tj > *Prp3* RNAi testes. We hypothesized that the cells accumulated in Tj > *Prp3* RNAi testes may be the undifferentiated germ cells without niche control. To test this hypothesis, we stained the testis and found that the undifferentiated cells could be labeled by anti-Vasa antibodies and accumulated without normal niche cells (Fig. 1D,E and Supplementary Fig. 1C,D). Moreover, only point fusomes existed (white arrowheads) and no branched fusomes were observed in Tj > *Prp3* RNAi testes (Fig. 1E”); however, branched fusomes (yellow arrowheads) were observed in differentiated germ cell cysts in the control testes (Fig. 1D”). We further stained with phosphor histone H3 (PH3), a proliferation marker, to test the cell fate of these undifferentiated germ cell cysts. These results showed that the undifferentiated germ cells in *Prp3* RNAi testes could proliferate and maintain themselves without hub cells (Fig. 1D,E and Supplementary Fig. 1C,D). Taken together, our data indicated that knockdown of *Prp3* gene by Tj-Gal4 could cause GSCs differentiation defects and tumor formation.

### Prp3 deficiency in spermatogonia causes spermatogonia differentiation defects

**Figure 2:**
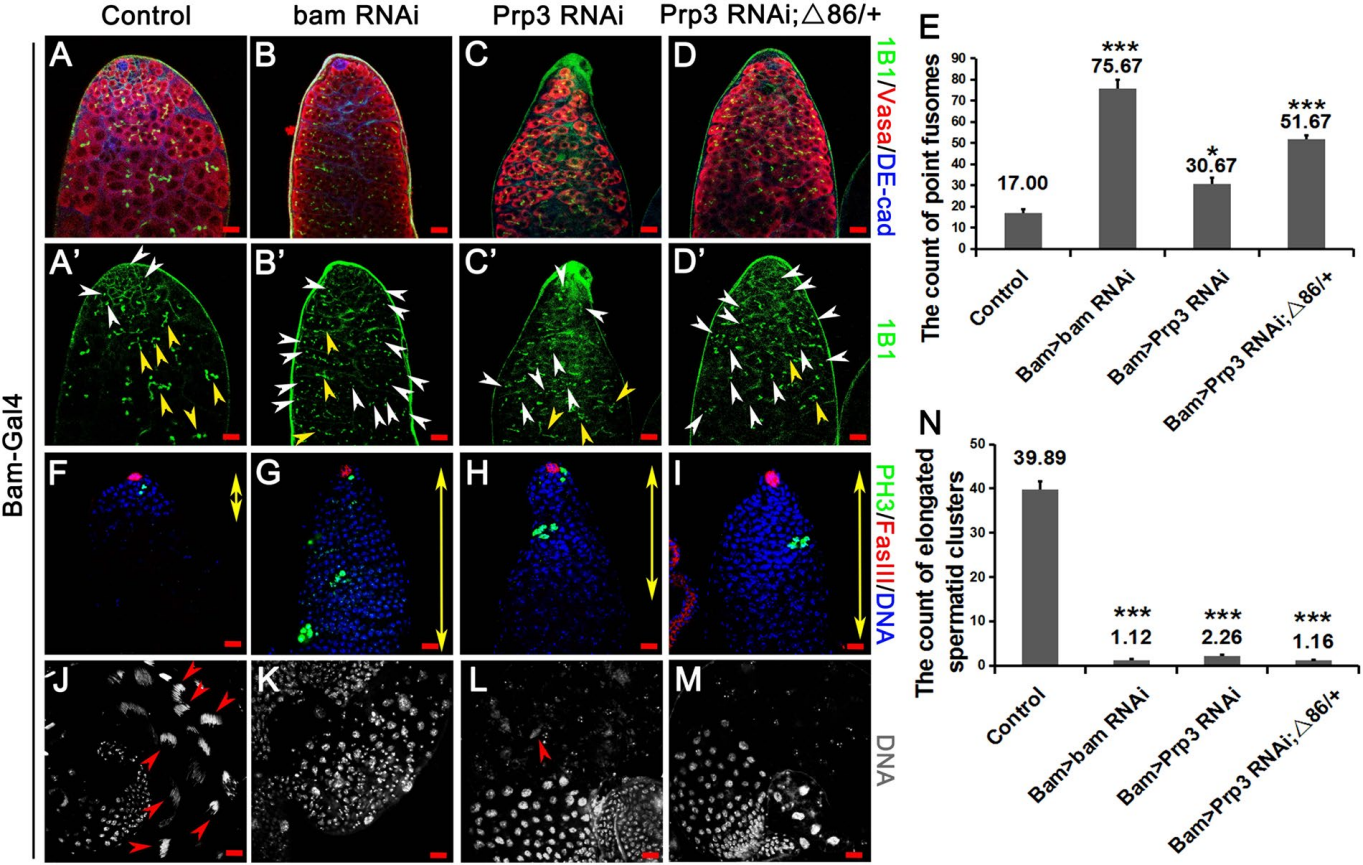
The function of Prp3 in spermatogonia. (**A**–**D**) Immunostaining of control, Bam > *bam* RNAi, Bam > *Prp3* RNAi, and Bam > *Prp3* RNAi; Δ86/+ testes. Representative point fusomes are indicated with white arrowheads and branch fusomes are indicated with yellow arrowheads. (**E**) The count of point fusomes in control, Bam > *bam* RNAi, Bam > *Prp3* RNAi, and Bam > *Prp3* RNAi; Δ86/+ testes. (**F**–**M**) Immunostaining of control, Bam > *bam* RNAi, Bam > *Prp3* RNAi, and Bam > *Prp3* RNAi; Δ86/+ testes. DNA was stained with Hoechst33342, and could label undifferentiated cells at the apex of the testis (yellow double arrowheads) and label the clusters of elongated spermatids at the tail of the testis (red arrowheads). (**N**) The count of elongated spermatid clusters. Data were analyzed by Student’s *t* test (all differences relative to control), *represents for P value < 0.05, **represents for P value < 0.01, ***represents for P value < 0.001. Error bars represent SEM. Scale bars: 20 µM.

Bam is a key differentiation factor in *Drosophila* testes. Knocking down of *bam* in spermatogonia severely affected germ cell differentiation. Notably, undifferentiated germ cells accumulated and obtained the ability to proliferate by themselves in Bam > *bam* RNAi testes (Fig. 2A,B,F,G).

We also questioned the role of Prp3 in spermatogonia differentiation. We next knocked down *Prp3* in spermatogonia driven by Bam-Gal4. In Bam > *Prp3* RNAi testes, point fusomes were significantly increased (Fig. 2A,C,E) with statistical differences (all differences relative to control), and PH3-positive cells could also be observed distant to the hub cells (Fig. 2F,H). Interestingly, *Prp3* mimicked the phenotype of *bam* in the spermatogonia transition from self-renewal to differentiation.

Previous study indicated that heterozygous mutation of *bam* did not lead to dramatic differentiation defects in *Drosophila* testes^7^. However, phenotype of differentiation defects were obviously enhanced in Bam > *Prp3* RNAi; Δ86/+ testes (more point fusomes and accumulated undifferentiated germ cells), compared with Bam > *Prp3* RNAi (Fig. 2C–E,H,I).

**Figure 3:**
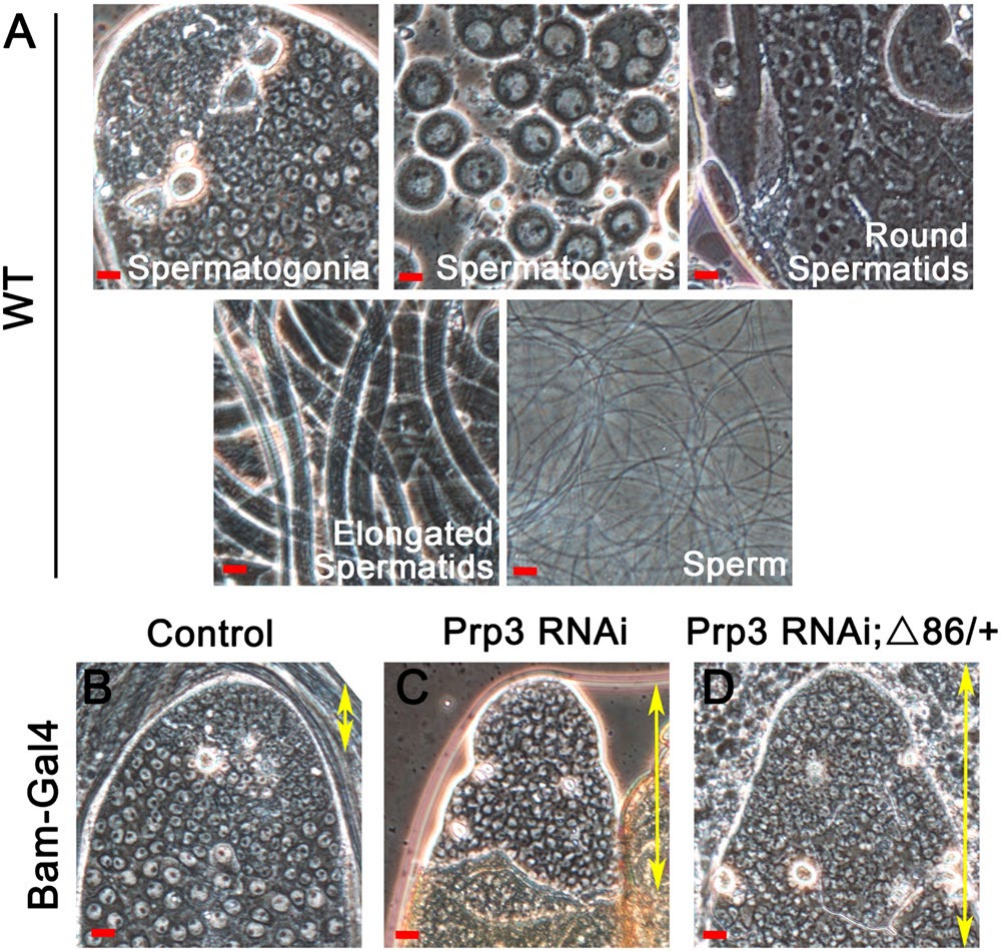
Knockdown of *Prp3* in spermatogonia caused germ cell differentiation defects. (**A**) Phase contrast view of wild-type testes. Spermatogenic cells at different stages (spermatogonia, spermatocytes, round spermatids, elongated spermatids, and mature sperm) could be identified. (**B**–**D**) Phase contrast view of the tip of the testis. Spermatogonia (yellow double arrowheads) accumulated in *Prp3* RNAi and *Prp3* RNAi; Δ86/+ testes compared with the control. Scale bars: 20 µM.

In control testes, many clusters of elongated spermatids (39.89 ± 1.80) could be observed, while only few clusters of elongated spermatids were observed in Bam > *Prp3* RNAi (2.26 ± 0.45), Bam > *bam* RNAi (1.12 ± 0.14), and Bam > *Prp3* RNAi; Δ86/+ (1.16 ± 0.18) testes (Fig. 2J–N, all differences relative to control). Phase-contrast view allowed us to identify spermatogenic cells at different stages (spermatogonia, spermatocytes, round spermatids, elongated spermatids, and mature sperm) in wild-type testes (Fig. 3A). Nonetheless, only spermatogonia existed and accumulated at the tip of testes in Bam > *Prp3* RNAi and Bam > *Prp3* RNAi; Δ86/+ cells compared with those of the control (Fig. 3B–D). These results suggested that the differentiation disorder of early germ cells caused by loss of Prp3 influenced spermatogenesis, ultimately leading to male infertility.

### Prp19 and Prp8 are essential for GSC self-renewal and differentiation in the *Drosophila* testis

**Figure 4:**
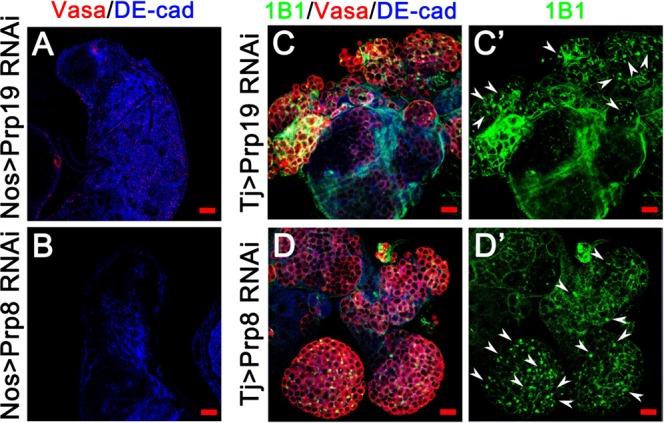
The phenotype of *Prp19* RNAi and *Prp8* RNAi in the *Drosophila* testis. Immunostaining of *Prp19* RNAi (**A**,**C**) and *Prp8* RNAi (**B**,**D**) testes. Representative point fusomes are indicated with white arrowheads. Scale bars: 20 µM.

The Prp complex is a large protein family that participates in RNA splicing. We next tested several other components of the Prp complex, and found that Prp19 and Prp8 have similar functions in *Drosophila* testes. When we knocked down *Prp19* and *Prp8* using Nos-Gal4, males were totally sterile (n = 63 for *Prp19*; n = 58 for *Prp8*). Moreover, males were totally sterile in Tj > *Prp19* RNAi (n = 44) flies and partially infertile in Tj > *Prp8* RNAi (10.53% fertile, n = 57) flies (Supplementary Table S1). DE-cad labels hub cells and cyst cells, and Vasa labels germ cells in the testis. For the abnormal structure of testes, germline stem cells adjacent to the hub cells and subsequently differentiated germ cells lost. After knockdown of *Prp19* and *Prp8* with Nos-Gal4, normal structure of testes disappeared (Supplementary Table S2), and Vasa-positive germ cells were absent (Fig. 4A,B) compared with those of the control (Fig. 1B). Moreover, only point fusomes existed among undifferentiated germ cells in Tj > *Prp19* RNAi and Tj > *Prp8* RNAi testes (Fig. 4C,D) when comparing with the control testes (Fig. 1D). These data suggested that *Prp19* and *Prp8* are also essential for GSC self-renewal and differentiation in fly testes.

### Prp3 regulates proliferation and cell survival in *Drosophila* S2 cells

**Figure 5:**
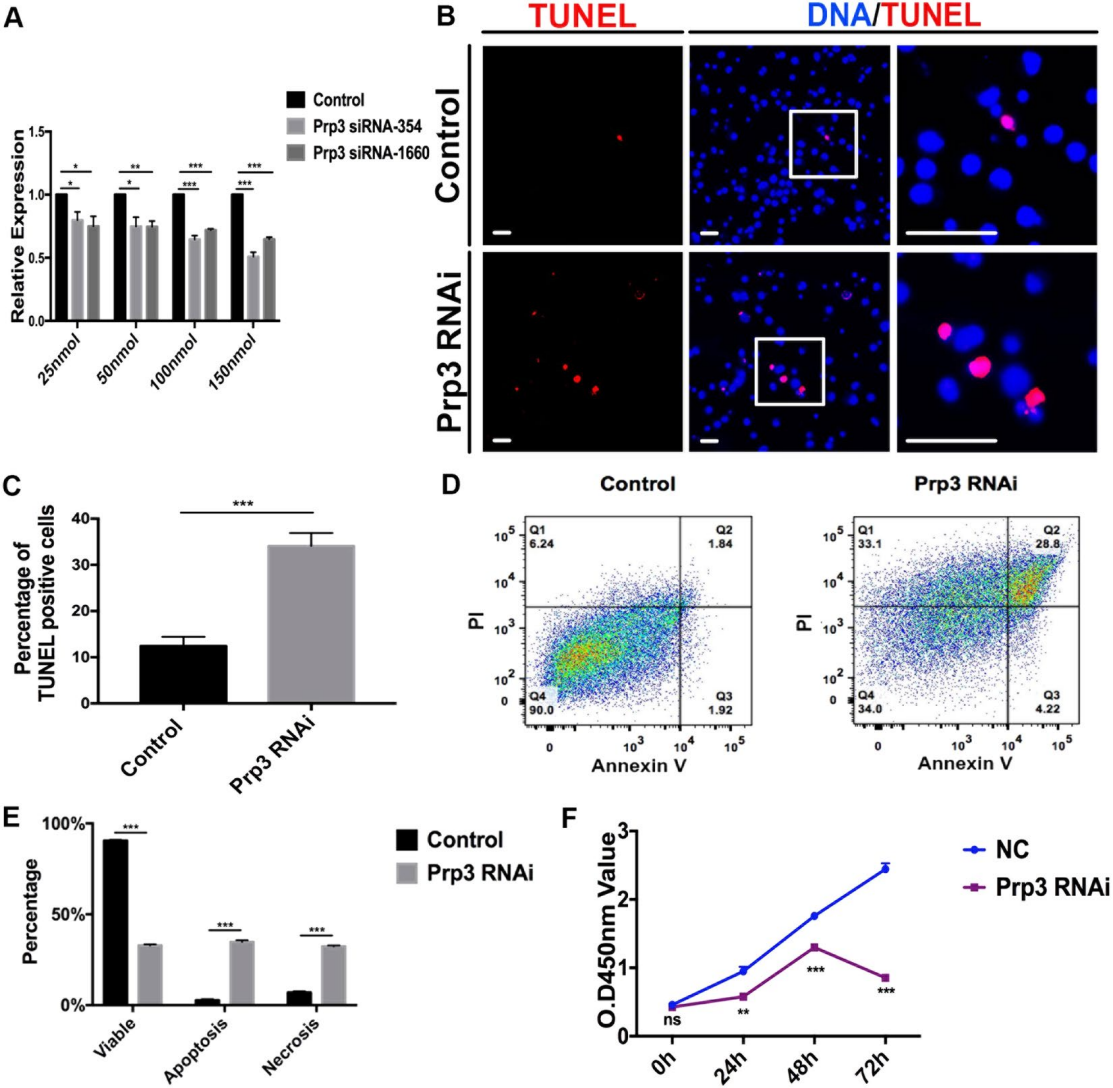
*Prp3* knockdown caused cell death in *Drosophila* S2 cells. (**A**) Relative *Prp3* mRNA level in control and *Prp3* siRNA (*Prp3* siRNA-354 and *Prp3* siRNA-1660) cells to validate knockdown efficiency. *Prp3* siRNA-354 was more efficient and used in following experiments. (**B**) Immunostaining of control and *Prp3* RNAi using TUNEL and DNA (Hoechst). (**C**) Percentage of TUNEL-positive cells in control and *Prp3* RNAi. (**D**) Flow cytometry testing of control and *Prp3* RNAi. Ratio of live (Q4), apoptotic (Q2 + Q3) and necrotic (Q1) cells were calculated at different intervals. (**E**) Percentage of cell components in control and *Prp3* RNAi. (**F**) CCK-8 assay for control and *Prp3* RNAi. Student’s *t* test was used for the statistical analysis. *represents for P value < 0.05, **represents for P value < 0.01, ***represents for P value < 0.001. Error bars represent SEM. Scale bar: 30 μM.

To explore the function of Prp3 in apoptosis and proliferation, we further downregulated *Prp3* expression using two siRNAs (*Prp3* siRNA-354 and *Prp3* siRNA-1660) by *in vitro* approaches. Our results indicated that siRNA-mediated knockdown of *Prp3* in *Drosophila* S2 cells reduced the expression of the *Prp3* mRNA (Fig. 5A).

In S2 cells, PH3-positive cells were significantly decreased in *Prp3* siRNA (150 nmol) S2 cells (Supplementary Fig. S2). Moreover, when *Prp3* was knocked down in these cells using *Prp3* siRNA (150 nmol), TUNEL-positive cells were dramatically increased (Fig. 5B,C), indicating that Prp3 was essential for cell survival. Similar results were obtained by flow cytometry, which showed that the ratios of apoptotic and necrotic cells significantly increased in *Prp3* siRNA S2 cells (Fig. 5D,E). Next, we used a CCK-8 kit to detect whether the growth of *Prp3* siRNA-treated S2 cells was affected, and found that knockdown of *Prp3* in S2 cells dramatically reduced cell proliferation, compared with control (Fig. 5F). Taken together, these results indicated that the *Prp3* knockdown results in decreased proliferation and increased cell death in *Drosophila*.

### Prp3 regulates the expression level of spliceosome subunits in *Drosophila* S2 cells

**Figure 6:**
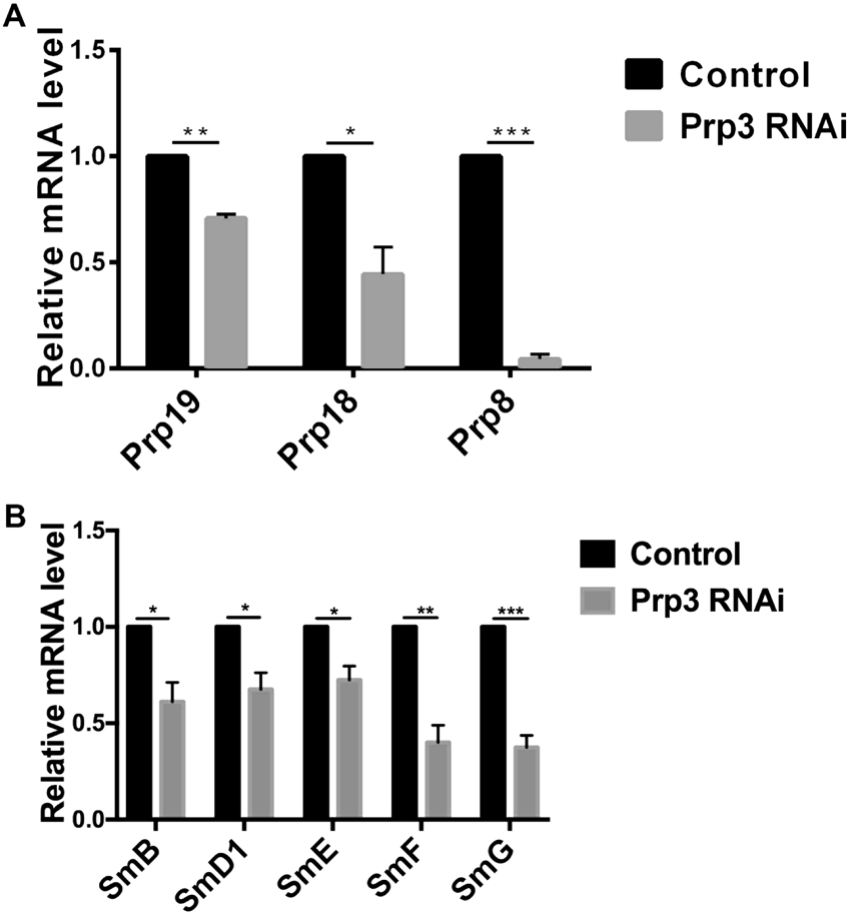
*Prp3* regulated the expression level of major spliceosome subunits. (**A**) Relative mRNA level of key subunits of Prp complex (*Prp19*, *Prp18*, *Prp8*) in control and *Prp3* RNAi cells. (**B**) Relative mRNA level of key subunits of Sm complex (*SmB*, *SmD1*, *SmE*, *SmF*, *SmG*) in control and *Prp3* RNAi cells. Student’s *t* test was used. *represents for P value < 0.05, **represents for P value < 0.01, ***represents for P value < 0.001. Error bars represent SEM.

To further investigate whether Prp3 affects spliceosome, we measured the expression level of major spliceosome subunits of the Prp complex and Sm complex. Surprisingly, the qRT-PCR results showed that spliceosome subunits, including key components of the Prp complex (*Prp18*, *Prp19*, and *Prp8*) and Sm complex (*SmB*, *SmD1*, *SmE*, *SmF*, and *SmG*), were all downregulated in *Prp3* siRNA S2 cells (Fig. 6). These results indicated that Prp3 may be a key protein that could regulate the expression level of spliceosome subunits.

## Discussion

The spliceosome is a fundamental element for the constitutive and alternative splicing of pre-mRNA to generate mature mRNA. The U4/U6-associated splicing factor, Prp3, is conserved in humans and *Drosophila* with 45% sequence identity. Prp3 is predominantly expressed in *Drosophila* ovaries and localizes in the nuclei of the female reproductive cells^31^. The other spliceosomal gene *prp22* (pea) is required for chromatin dispersal in nurse cell nuclei during oogenesis^32^. However, the biological function of Prp3 in male fertility and stem cell niche remain to be determined.

Here, we systematically analyzed the mechanism and function of Prp3 in *Drosophila* using *in vivo* and *in vitro* approaches. Our results indicated that Prp3 plays key roles for the germline stem cell niche in the *Drosophila* testis, and controls the GSC self-renewal and differentiation processes. Our data provided a model of Prp3 which functions in the germline stem cell niche, that maintenance defects of GSCs caused abnormal structure of testes and loss of germ cells, while maintenance defects of CySCs led to dysfunction of somatic stem cells and, followed by early germ cell differentiation defects with non-cell autonomous function.

The S2 cell line was derived from a primary culture of late stage (20–24 hours old) *Drosophila melanogaster* embryos. Although the S2 cell line is not a germ cell line, but it is a classical cell model in *Drosophila*. Knockdown of *Prp3 in vitro* decreased the proliferation ability and dramatically increased the cell death ratio in S2 cells, which imitated the phenotype in the germline stem cell niche. More importantly, *in vitro* assays provided evidence that knockdown of *Prp3* might destroy the major structure of the spliceosome and affect its function by regulating major subunits of the Prp and Sm complexes. Although there are subtle differences in some biological events, the evidence indicated that the spliceosome complex may play critical roles in azoospermia and germ cell tumor formation. Further assessment using a germline stem cell model will be investigated in future studies.

By querying The *Drosophila* Interactions Database (DroID), we found that Prp3 could bind with many snRNPs and Prp factors, such as SmD2, LSm3, U2af50, and U4-U6-60K^33^. Moreover, the Prp19 complex regulates the ubiquitination modification of Prp3^34^. On the other hand, Prp8 can also recognize the ubiquitination chains of Prp3 and stabilizes the U4/U6.U5 tri-snRNP^35^.

In our study, we observed that Prp3, Prp8, and Prp19 played diverse roles in GSCs and CySCs. GSCs lacking *Prp3*, *Prp8*, and *Prp19* could not maintain themselves while CySCs lacking *Prp3*, *Prp8*, and *Prp19* are not sufficient for GSCs to differentiate to terminal germ cells. We hypothesized that this is caused by diverse splicing of pre-mRNA targets. However, the effector functions of Prp3, Prp8, and Prp19 in GSCs and CySCs remain poorly understood. Screening for the downstream factors and splicing targets of Prp3, Prp8, and Prp19 in the germline stem cell niche will provide new insights for understanding the mechanism of azoospermia and germ cell tumor formation.

## Materials and Methods

### Fly strains

All flies were cultured on standard corn meal food at 25 °C^7^. Information for alleles or transgenic lines used in this study can be found as noted: Nos-Gal4 (BDSC, #4937), Tj-Gal4 (DGRC, #104055), UAS-*Prp3* RNA interference (RNAi) (THFC, #TH02381.N), UAS-*Prp8* RNAi (THFC, #THU1528), UAS-*Prp19* RNAi (THFC, #THU1054), UAS-*bam* RNAi (THFC, #THU0567). Bam-Gal4; △86/+ line is a gift from DH Chen. The W^1118^ line is used as wild type fly.

### Fly crosses

We used UAS/GAL4 system to knock down shRNA-targeted genes in specific cell populations. All UAS-RNAi transgenic lines were obtained from the THFC^29^. GAL4 males are crossed with UAS-RNAi virgin females raised at 25 °C. In the next generation, we selected males with both GAL4 and UAS-RNAi elements.

### Cell culture and transfection

*Drosophila* Schneider 2 (S2) cells were grown at 28 °C in Schneider’s medium (21720024, Gibco, USA) supplemented with 10% heat-inactivated fetal bovine serum (FBS) (04-001-1ACS, Bioind, Israel)^36^. For knockdown of *Prp3*, S2 cells were transfected using Lipofectamine 2000 (Lipo2000; 11668019, Invitrogen, USA). siRNAs were designed and synthesized by GenePharma company (Suzhou, China). Detailed information is as follows: negative control, F: 5-UUCUCCGAACGUGUCACGUTT-3, R: 5-ACGUGACACGUUCGGAGAATT-3; *Prp3* siRNA-1660, F: 5-GCUCAUGCUAACGCGCAUUTT-3, R: 5-AAUGCGCGUUAGCAUGAGCTT-3; *Prp3* siRNA-354, F: 5-GCAAGCGGGCCCUGAGUAATT-3, R: 5-UUACUCAGGGCCCGCUUGCTT-3.

### Immunofluorescence

Fly testes were dissected and fixed for 30 min in 4% paraformaldehyde. After washing three times in 1x PBS with 0.1% Triton X-100 (PBST) and blocking for 1 hr in 5% bovine serum albumin, samples were incubated with primary antibodies overnight at 4 °C. After washing three times for 10 min in 0.1% PBST, the samples were incubated for 1 hr with secondary antibodies at room temperature followed by three times washing in 0.1% PBST. Testes were then stained with Hoechst 33342 (1.0 mg/ml, Invitrogen) for 5 min before mounting. S2 cells were cultured for 24 hours, and immunostaining was carried out in the culture dish according to the protocols described above^7^.

The antibodies used were as follows: mouse anti-FasIII (Developmental Studies Hybridoma Bank [DSHB], 1:50); mouse anti-Eya (DSHB, 1:50); rat anti-DE-cadherin (DSHB, 1:20); mouse anti-1B1 (DSHB, 1:75); rabbit anti-Vasa (1:1000, Santa Cruz); rabbit anti-PH3 (Cell Signaling Technology [CST], 1:400); rat anti-Zfh1 (1:2000, a gift from C Tong). Secondary antibodies conjugated to A488, Cy3, A594, or A647 (Molecular Probes and Jackson Immunologicals) were diluted at 1:1000.

### Male fertility test

Single male fertility test was performed by using a single F1 RNAi adult male fly enclosed for three days in a cross with three wild type virgin females at room temperature^8^.

### Phase contrast view

Fly testes were dissected in 1x PBS and washed several times. Testes were observed on slides by a phase-contrast microscope after gently squashing them with a cover slip^7^.

### Quantitative reverse transcription RT-polymerase chain reaction (qRT-PCR)

Total RNA was extracted using Trizol reagent (9108, Takara, Japan). cDNA was synthesized using Prime Script RT Reagent Kit (RR037A, Taraka, Japan), and qRT-PCR was performed by using SYBR Premix Ex Taq (RR420A, Takara, Japan). GAPDH was amplified as an internal standard. Fold changes were calculated using the standard curve according to the manufacturer’s protocol^36^. Each experiment was independently repeated three times. All primers used for qRT-PCR are listed in Supplementary Table S3.

### Terminal deoxynucleotidyl transferase-mediated dUTP-biotin nick-end labeling (TUNEL) assay

S2 cells were collected after transfection for 48 h. Cell apoptosis was determined using the TUNEL assay according to the manufacturer’s protocols^37^. The TUNEL BrightRed Apoptosis Detection Kit was obtained from Vazyme (A113, Nanjing, China).

### Flow cytometry assay

S2 cells were collected after transfection for 48 h. Flow cytometry was performed using an Annexin V/PI Apoptosis Assay Kit (FMSAV647-100, FcMACS, Nanjing, China)^37^. After transfection for 48 h, S2 cells were washed with ice-cold PBS. Different cell groups were stained with the apoptosis detection kit according to the manufacturer’s instructions. The samples were then analyzed by using FACScan flow cytometry (BD Biosciences, San Jose, CA, USA).

### Cell Counting Kit-8 (CCK-8) assay

CCK-8 assay (CK04-3000T, DOJINDO, Japan) was utilized to assess *Drosophila* S2 cell viability according to the manufacturer’s protocols^37^. Briefly, transfected S2 cell were transferred to 96-well plates (3000 cells per well), and incubated in 10% CCK-8 reagent that was diluted in Schneider’s medium at 37 °C for 1 h. After transfected at 0 h, 24 h, 48 h and 72 h, the absorbance in each well was evaluated at 450 nm (Multiskan GO, Thermo Scientific, Waltham, USA).

### Statistical analysis

The quantitative results are presented as mean ± standard error of mean (SEM), and the data were evaluated for statistical differences using student’s *t*-test and one-way ANOVA by Graphpad software (https://www.graphpad.com/) or Microsoft Excel. Chi-square test was used to evaluate for ratio results. *P < 0.05; **P < 0.01; ***P < 0.001.

## Supplementary information

Supplementary file
