# Supplementary material for: Precursor RNA processing 3 is required for male fertility, and germline stem cell self-renewal and differentiation via regulating spliceosome function in Drosophila testes
Source: Sci Rep. 2019 Jul 10;9:9988. doi: 10.1038/s41598-019-46419-x (PMC6620278; doi:10.1038/s41598-019-46419-x)
Supplement: Supplementary file 1 — Supplementary file [file 41598_2019_46419_MOESM1_ESM.docx]

**Precursor RNA processing 3 is required for male fertility, and germline stem cell self-renewal and differentiation via regulating spliceosome function in *Drosophila* testes**

Xia Chen^1, 2, #^, Xiaojin Luan^1, #^, Qianwen Zheng^1, 2, #^, Chen Qiao^3, #^, Wanyin Chen^1^, Min Wang^1^, Yidan Yan^1^, Bing Xie^1^, Cong Shen^4^, Zeyu He^5^, Jun Zhang^6^, Mingxi Liu^6^, Xing Hu^1^, Hong Li^4^, Bo Zheng^4, 6, *^, Jie Fang^1, *^, Jun Yu^1, 2, 6, *^

^1^Department of Gynecology, the Affiliated Hospital of Jiangsu University, Jiangsu University, Zhenjiang Jiangsu 212001, China

^2^Reproductive Sciences Institute of Jiangsu University, Jiangsu University, Zhenjiang Jiangsu 212001, China

^3^Department of Clinical Pharmacy, the Affiliated Hospital of Jiangsu University, Jiangsu University, Zhenjiang Jiangsu 212001, China

^4^Center for Reproduction and Genetics, Suzhou Municipal Hospital, the Affiliated Suzhou Hospital of Nanjing Medical University, Suzhou Jiangsu 215002, China

^5^Department of Clinical Medicine, China Medical University, Shenyang Liaoning 110001, China

^6^State Key Laboratory of Reproductive Medicine, Department of Histology and Embryology, Nanjing Medical University, Nanjing 211166, China

^#^These authors contributed equally to the work.

^*^Correspondence and requests for materials should be addressed to B.Z. (email: mansnoopy@163.com), J.Y. (email: yujun9117@126.com), J.F. (email: fangjie070@163.com).

**Supplementary information**

**Supplementary Figure S1**

**
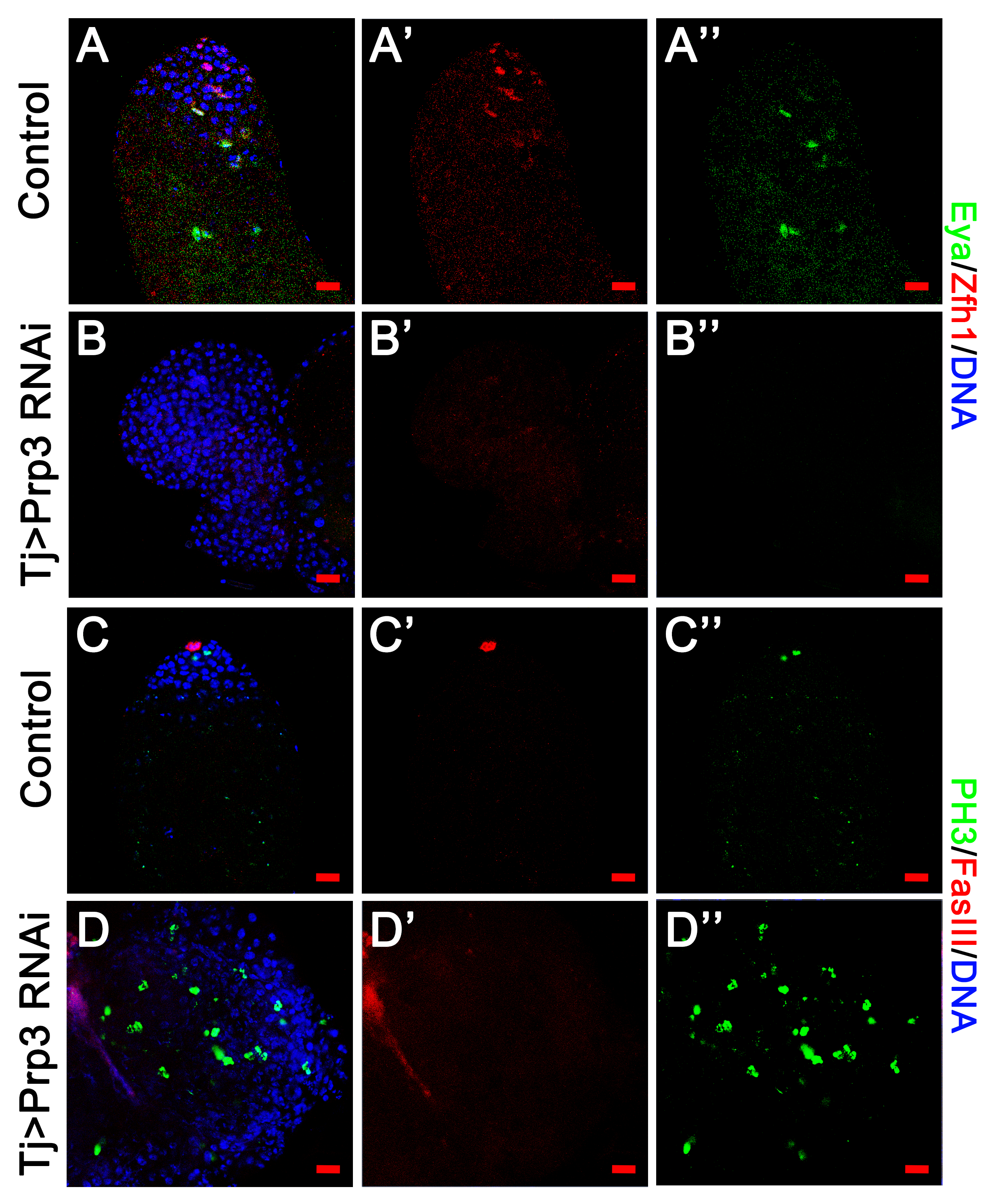
**

**Supplementary Figure S1.** The phenotype of *Prp3* knockdown driven by Tj-Gal4 in *Drosophila* testes. Immunostaining of control and *Prp3* RNAi cells using anti-Zfh1 (red in A-B), anti-Eya (green in A-B), anti-FasIII (red in C-D), and anti-PH3 (green in C-D). DNA was stained with Hoechst33342. Scale bars: 20 µM.

**Supplementary Figure S2**

**
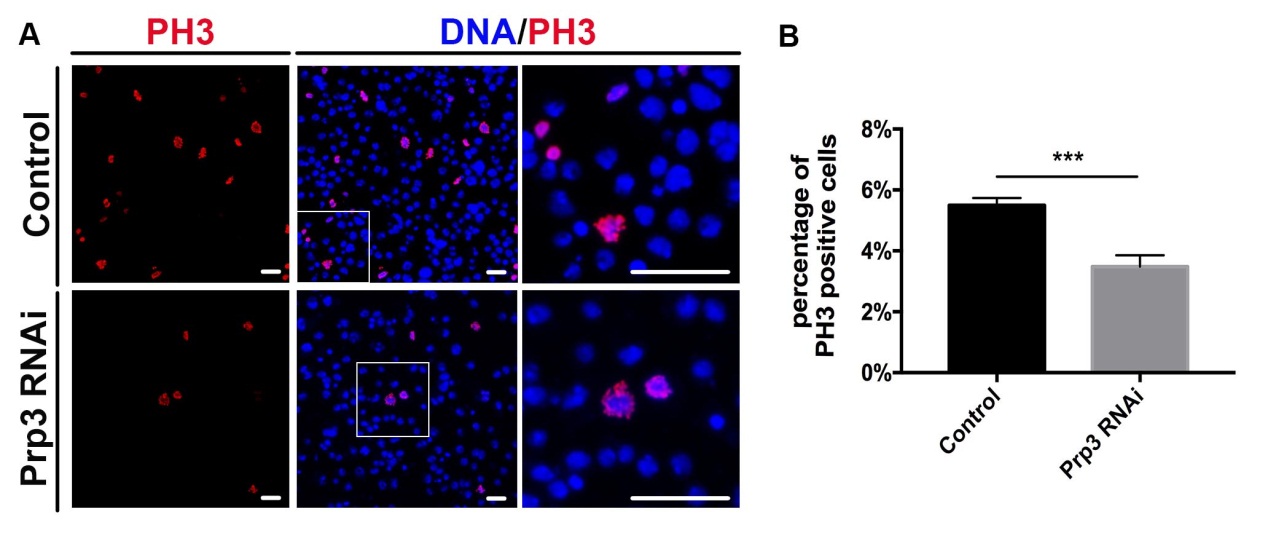
**

**Supplementary Figure S2.** *Prp3* knockdown reduced proliferation ability in *Drosophila* S2 cells. (A) Immunostaining of control and *Prp3* RNAi cells using anti-PH3 (red) and DNA (Hoechst). (B) Percentage of PH3-positive cells in control and *Prp3* RNAi. Student’s *t* test was used. ****P*< 0.001. Error bars represent SEM.

**Supplementary Table S1.** Fertility rate of *Prp19* and *Prp8* RNAi flies.

|  | Fertile lines  /Total lines | Male fertility rate, % | *P* value |
| --- | --- | --- | --- |
| W^1118^ | 86/89 | 96.63% |  |
| Nos>Prp19 RNAi | 0/63 | 0.00% | *** |
| Nos>Prp8 RNAi | 0/58 | 0.00% | *** |
| Tj>Prp19 RNAi | 0/44 | 0.00% | *** |
| Tj>Prp8 RNAi | 6/57 | 10.53% | *** |
| Chi-square test was used. ***, *P*<0.001 | | | |

**Supplementary Table S2.** The ratio of testes with normal structure.

|  | Testes with normal structure/Total testes | Ratio | *P* value |
| --- | --- | --- | --- |
| Control | 56/56 | 100.00% |  |
| Nos>Prp3 RNAi | 0/68 | 0.00% | *** |
| Nos>Prp19 RNAi | 0/49 | 0.00% | *** |
| Nos>Prp8 RNAi | 0/44 | 0.00% | *** |
| Chi-square test was used. ***, *P*<0.001 | | | |

**Supplementary Table S3.** The realtime primer sequences used in this study.

| **Gene** | **Forward primer(5'-3')** | **Reverse primer(5'-3')** |
| --- | --- | --- |
| Prp3 | TGGAAGGAGGAGCAGGAGAAGATC | AGCACACGCATCAGGTTGGATATG |
| Prp19 | GCTGCCACGAAGGACCTGTTAC | CCTGTGCGGATATCGGAGAATGC |
| Prp18 | GCTGCTCACCTTCCTGCTCAAG | AGCGGCTTAACGTATTCCTTAGTCTG |
| Prp8 | GCAGGAGAGGCAGCACAACTAC | AGCGACGTGATTCCAGCCAATG |
| SmB | CATGAACTTGATCCTCGGCGACTG | CCTCTGGCGGCGGTGGTC |
| SmD1 | CACCTGAAGAGCGTTCGGATGAC | TGTCGTCGATGAGGAGCGTCTC |
| SmE | CCATCAACCTGATCTTCCGTTACCTG | GCGTCGTCCAGCACCAGATTC |
| SmF | GCTCCGTGACTGGTAATCTTGGC | TCCTCGTCGTCGTCCTCCATG |
| SmG | TCTGAGCAGCGTGGTTATTGACATC | CGTAGCATGGCATCGAGTCCTTG |
| GAPDH | GTGGTGAACGGCCAGAAGAT | GCCTTGTCAATGGTGGTGAA |
